# Wavelength Tunable Pulsed Lasers Enabled by a Versatile Metafiber Functioning as Both Saturable Absorber and Filter

**DOI:** 10.1002/advs.202511572

**Published:** 2025-10-17

**Authors:** Bo Fu, Chenxi Zhang, Zhouqi Zhang, Zuxi Ouyang, Gang Wang, Xiuhan Jing, Weilin Chen, Lei Zhang, Min Qiu

**Affiliations:** ^1^ Hangzhou International Innovation Institute Beihang University Hangzhou 311115 China; ^2^ Key Laboratory of Precision Opto‐Mechatronics Technology of Education Ministry School of Instrumentation and Optoelectronic Engineering Beihang University Beijing 100191 China; ^3^ Key Laboratory of 3D Micro/Nano Fabrication and Characterization of Zhejiang Province School of Engineering Westlake University 18 Shilongshan Road Hangzhou Zhejiang Province 310024 China; ^4^ Institute of Advanced Technology Westlake Institute for Advanced Study 18 Shilongshan Road Hangzhou Zhejiang Province 310024 China; ^5^ Photonic Systems Laboratory (PHOSL) École Polytechnique Fédérale de Lausanne (EPFL) Lausanne 1015 Switzerland; ^6^ QianYuan National Laboratory HangZhou 310024 China; ^7^ Westlake Institute for Optoelectronics Fuyang Hangzhou 311421 China

**Keywords:** Fabry‐Perot interferometer, metafiber, Q‐switching, tunable filter, wavelength‐tunable pulsed laser

## Abstract

Wavelength‐tunable pulsed lasers have garnered significant research interest due to their critical role in applications requiring precise spectral matching, such as wavelength‐division multiplexing and spectroscopic analysis. Although recent hybrid architectures integrating saturable absorbers with wavelength‐selective components have enabled notable progress, these systems remain constrained by two fundamental challenges: excessive coupling losses and complex alignment procedures. Here, a compact wavelength‐tunable pulsed laser configuration is presented that employs a monolithic fiber‐based component, effectively addressing the complexity and alignment issues inherent in conventional hybrid systems. By leveraging advanced manufacturing techniques, a 2D metafiber is integrated with a 3D Fabry‐Perot interferometer at the end facet of a single‐mode optical fiber, forming a metafiber Fabry‐Perot structure that simultaneously functions as both a saturable absorber and a tunable optical filter. Exploiting the versatile optical feedback provided by the Fabry‐Perot cavity, our design achieves Q‐switching operation in the telecommunication band and enables approximately 10 nm dynamic wavelength tuning through temperature/refractive index modulation. This work circumvents the structural complexity of traditional hybrid systems while ensuring stable laser operation, demonstrating a promising pathway for high‐performance wavelength‐tunable pulsed laser applications.

## Graphical abstract

Schematic diagram of wavelength‐tunable pulsed laser based on metafiber‐FP (Fabry‐Perot). The alcohol solution is filled inside the FP cavity to simultaneously form a spectral filter and a saturable absorber. By adjusting the refractive index value and temperature inside the cavity, the tunable filtering effect is introduced to realize wavelength‐tunable pulsed laser generation.

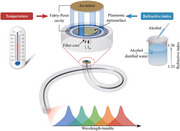

## 1 Introduction

Pulsed lasers, distinguished from continuous‐wave counterparts by their ability to manipulate key parameters such as peak power, pulse duration, and repetition rate, have found extensive applications across diverse fields.^[^ ^1^ ^]^ Among pulsed laser technologies, Q‐switching stands out as a primary method for generating stable high energy pulsed outputs. By modulating the cavity quality factor, this technique compresses energy into microsecond‐duration pulses, achieving peak powers that exceed the average output by three orders of magnitude.^[^ ^2^ ^]^ Such characteristic renders the Q‐switched lasers well‐suited for applications like precision laser ablation in materials processing^[^ ^3^, ^4^ ^]^ and minimally invasive surgical procedures.^[^ ^5^, ^6^ ^]^ The pursuit of efficient pulse generation has spurred innovation in saturable absorber (SA) materials. Traditional carbon‐based SAs offer broadband operation but suffer from limited damage thresholds.^[^ ^7^, ^8^ ^]^ Transition metal dichalcogenides provide wavelength‐selective absorption but exhibit slower recovery times.^[^ ^9^ ^]^ Topological insulators combine ultrafast recovery with relatively high modulation depth, yet their integration into the laser systems remains challenging.^[^ ^10^ ^]^ The latest breakthroughs involve plasmonic metasurfaces, which are designed subwavelength nanostructures to achieve extraordinary optical properties in both linear and nonlinear regimes.^[^ ^11^, ^12^, ^13^ ^]^ Specifically, metasurfaces enable independent manipulation of polarization, phase, amplitude, and even the frequency of electromagnetic waves at nanoscale,^[^ ^14^ ^]^ advancing the applications in beam shaping,^[^ ^15^ ^]^ sensing^[^ ^16^ ^]^ and nonlinear optics,^[^ ^17^ ^]^ to name a few. Integrating the plasmonic metasurfaces at the end facet of single‐mode optical fibers (forming “metafibers”), recent studies demonstrated pulsed fiber lasers operating in mode‐locked and Q‐switched region.^[^ ^18^, ^19^ ^]^ Benefitting from precise geometry and periodicity of the unit cell, plasmonic metafibers exhibiting outstanding saturable absorption closely related to the plasmonic resonances. Nevertheless, despite their versatility in tailoring light fields spatially and resonating at designed wavelengths, metasurfaces or metafibers typically exhibit fixed, time‐invariant responses post fabrication.

Meanwhile, wavelength‐tunable pulsed lasers have attracted growing research attention for applications requiring precise spectral matching, such as biomedical imaging^[^ ^20^, ^21^ ^]^ wavelength‐division multiplexing^[^ ^22^ ^]^ and spectroscopy analysis.^[^ ^23^, ^24^ ^]^ Conventional tunable systems typically rely on hybrid architectures combining SAs with wavelength‐selective components. These hybrid configurations achieve spectral agility through two primary mechanisms by either introducing spectral filtering^[^ ^25^, ^26^ ^]^ into the fiber laser cavity, or leveraging nonlinear polarization rotation.^[^ ^27^, ^28^ ^]^ While these methods deliver tuning ranges up to tens of nanometers with microsecond switching speeds,^[^ ^29^, ^30^ ^]^ they face critical limitations‐both approaches require complex integration of fiber circuits with discrete optical/electrical filters, leading to inevitable coupling losses and alignment challenges.^[^ ^30^ ^]^ Additionally, filtering configurations are constrained by fixed spectral responses due to their limited resonance band, whereas polarization‐based systems are highly susceptible to environmental perturbations because of their reliance on polarization dynamics.^[^ ^25^ ^]^ These trade‐offs between performance and practicality highlight the need for novel designs to balance simplicity and stability. Alternatively, an emerging direction combining the 2D metasurfaces and 3D Fabry‐Perot (FP) cavities,^[^ ^31^, ^32^, ^33^ ^]^ can offer additional degrees of freedom for dynamic resonance tuning, which may provide an effective solution.

**Figure 1:**
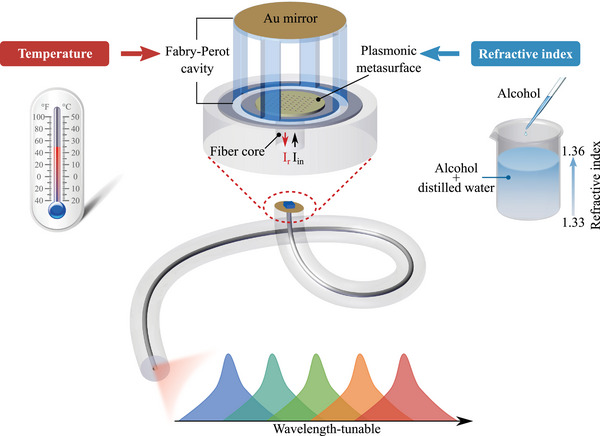
Schematic diagram of wavelength‐tunable pulsed laser based on metafiber‐FP. The alcohol solution is filled inside the FP cavity to simultaneously form a spectral filter and a saturable absorber. By adjusting the RI value and temperature inside the cavity, the tunable filtering effect is introduced to realize wavelength‐tunable pulsed laser generation.

Here, we propose and demonstrate a wavelength‐tunable pulsed laser using a single, compact fiber component, circumventing the complexity and alignment issues inherent in traditional hybrid systems. This fiber component features a hybrid‐2D‐metasurface‐and‐3D‐FP configuration that is integrated at a single‐mode fiber end facet by our newly developed fabrication method.^[^ ^32^ ^]^ The hybrid configuration consists of three layers: a gold plasmonic metasurface (metafiber)^[^ ^18^, ^34^ ^]^ served as the bottom reflector, a middle polymer‐based FP open cavity and a top gold film forming the second reflector. The whole configuration is termed as metafiber‐FP. As shown in **Figure** **1**, this metafiber‐FP simultaneously acts as an SA and a tunable optical filter. Leveraging the FP cavity's versatile optical feedback, our work establishes Q‐switching at the telecommunication band and then enables ∼10 nm wavelength tuning through temperature or refractive index (RI) modulation, eliminating the complexity of traditional hybrid systems while maintaining stable operation.

## 2 Results

### 2.1 Theoretical Model of Metafiber‐FP

**Figure 2:**
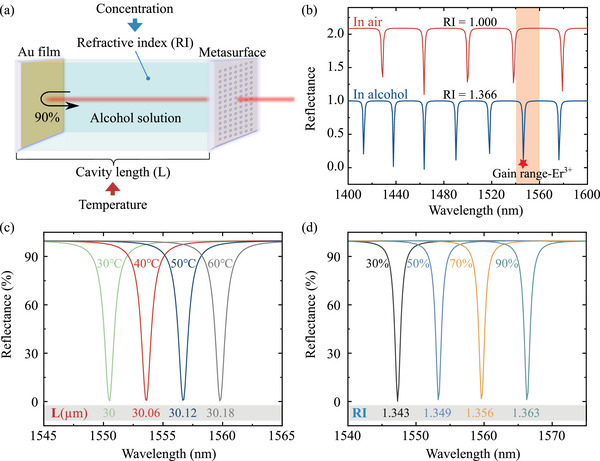
Simulation of reflectance spectral modulation in metafiber‐FP. a) Schematic of the simulation model. b) Reflectance spectra of the metafiber‐FP filled with air and alcohol, respectively. c) Trend of reflectance spectra of metafiber‐FP with cavity length tuning (via temperature control) control for resonance valley positioning in the Er^3 +^ gain band. d) Trend of the reflectance spectra of the metafiber‐FP with RI value running (via concentration control) for the resonance valley in the Er^3 +^ gain band.

In the metafiber‐FP configuration, an FP cavity is formed between the metasurface fabricated on the fiber end facet and a uniform gold film, which acts as a filter interferometer, as illustrated in **Figure** **2**a. In this study, the FP cavity is filled with an alcohol‐distilled water solution, and both the gold metasurface and gold film exhibit a reflectivity (*R*) of 90%. The transmission spectrum of the beam is described by the following equation:^[^ ^35^ ^]^

(1) $$T=\frac{{\left(1-R\right)}^{2}}{{\left(1-R\right)}^{2}+4\pi \sin^{2}{\left(\frac{\delta }{2}\right)}^{2}}$$

where δ represents the round‐trip phase shift of the beam, described as: δ=4πnL/λ. Here n denotes the RI value of the FP cavity medium, *L* is the physical length of the FP cavity, and λ is the wavelength of the incident light. By independently adjusting the temperature and concentration of the solution, we can separately modulate *L* and *n*, thereby realizing the spectral tuning within the FP cavity. Figure 2b shows the reflectance spectra when the FP cavity is filled with air and pure alcohol, respectively. The alcohol‐filled FP cavity exhibits an increased number of resonance peaks, with distinct high‐intensity peaks emerging within the gain interval of Er^3 +^. To realize a 1.5‐μm wavelength‐tunable pulsed laser, we focus on the resonance peaks within the Er^3 +^ gain region (highlighted in orange in Figure 2b) and implement wavelength tuning by adjusting *L* and *n*. First, temperature regulation provides effective control over the cavity length. As shown in Figure 2c, increasing the temperature of the liquid‐filled cavity from 30 to 60°C induces stepwise increases in cavity length in 0.06‐μm increments. This thermal tuning mechanism enables the resonant peaks to shift smoothly within the gain range from 1550.5 to 1559.8 nm. Additionally, modifying the alcohol concentration alters the solution's refractive index: increasing the volumetric concentration from 30% to 90% raises the RI from 1.343 to 1.363. As demonstrated in Figure 2d, this refractive index modulation shifts the resonance peak from 1547.3 to 1566.3 nm. Such numerical model clearly demonstrates that adjusting the temperature (to tune *L*) and refractive index (via concentration, to tune RI) enables precise modulation of the metafiber‐FP resonance peak positions. Subsequently, by incorporating this metafiber‐FP filter into the pulsed laser system and superimposing its spectral intensity with the gain curve, we achieve central‐wavelength‐tunable operation of the pulsed laser within the targeted Er^3 +^ gain band.

### 2.2 Theoretical Modeling of Wavelength‐Tunable Q‐Switched Pulsed Lasers

To comprehensively verify the frequency‐domain modulation characteristics of the metafiber‐FP for Q‐switched pulsed lasers, a numerical simulation investigation of Q‐switched pulses is carried out. This study relies on a coupled model that integrates the modified nonlinear Schrödinger equation and the rate equation. This integrated model is designed to simultaneously capture the temporal evolution of laser pulses and the spectral characteristics of the entire laser system, thereby providing a more accurate theoretical framework for the analysis. The coupled model fully incorporates the loss effects induced by gain filtering, applying corresponding energy‐dependent losses to photons at different energy levels within the Q‐switched pulse envelope.^[^ ^36^ ^]^ When computing the optical intensity, the contributions of photons across all energy levels are cumulatively integrated to synthesize the depletion gain profile. This methodological approach enables the realization of wavelength‐specific Q‐switched operation by selectively manipulating photon populations at the target wavelength. The underlying governing equations of the coupled model, as referenced in [37, 38] are presented as follows:

(2) $$i\frac{\partial A}{\partial z}=\frac{\beta_{2}}{2}\frac{\partial^{2}A}{\partial T^{2}}-\gamma \left|A^{2}\right|A+i\frac{1}{2}\frac{g_{0}}{1+E_{\mathrm{pulse}}/E_{\mathrm{sat}}}\left(1+T_{2}^{2}\frac{\partial^{2}}{\partial T^{2}}\right)A$$

(3) $$\frac{\partial g}{\partial t}=\frac{g_{0}-g}{\tau_{g}}-\frac{g|A^{2}|}{E_{\mathrm{sat}}}$$

**Figure 3:**
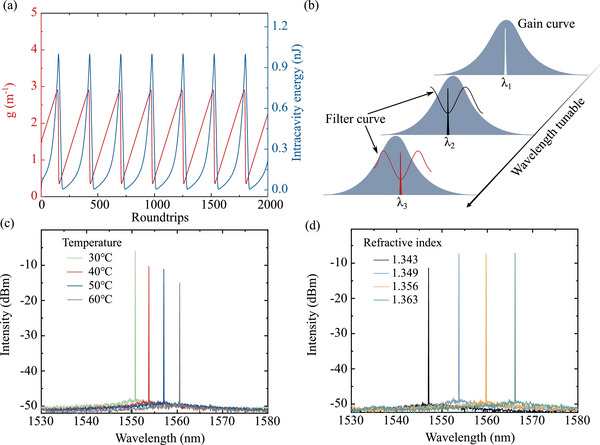
Simulation of wavelength‐tunable Q‐switched pulses employing metafiber‐FP trap filter. a) Schematic diagram of the wavelength‐tunable Q‐switched pulse generation system based on a tunable trap filter. b) Simulation of gain and intracavity energy modulation processes of Q‐switched pulsed lasers. c) Wavelength‐tunable spectra simulation via metafiber‐FP modulation spectra based on temperature control. d) Wavelength‐tunable spectra simulation via metafiber‐FP modulation under RI control.

Here, *A* represents the slowly varying amplitude envelope of the optical field, *z* denotes the propagation distance, *T* = *t* − *z*/ν_*g*_ is the group velocity reduced time coordinate, β_2_ characterizes the group velocity dispersion, *T*_2_ is the dipole relaxation time, γ is the nonlinear Kerr coefficient, *g* is the dynamic gain coefficient, *g*_0_ is the small‐signal gain coefficient, τ_*g*_ represents the dopant population relaxation time, and *E*_sat_ is the saturation energy. Equation (2) describes the spatiotemporal propagation of laser pulses in the fiber medium, explicitly accounting for dispersive, nonlinear, and gain/loss effects. Equation (3) governs the dynamic evolution of the gain coefficient, incorporating both pump‐induced gain recovery and intensity‐dependent gain saturation. Numerical simulations employ parameters closely aligned with experimental configurations. Specifically, the group velocity dispersion parameters are set to 22 ps^2^/km for the single‐mode fiber and +12 ps^2^/km for the gain fiber, reflecting their distinct chromatic dispersion characteristics. Key erbium‐doped fiber parameters include a population relaxation time of 10 ms, a small‐signal gain coefficient of 37 dB, and a saturation energy of 1 μJ, which are critical for accurately modeling the gain medium dynamics. Moreover, we have added comprehensive noise model by incorporating realistic noise sources to ensure that the simulations more accurately predict experimental data, including spontaneous emission noise, detector electronic noise, and intensity fluctuations caused by environmental disturbances.^[^ ^39^, ^40^ ^]^ The initial intracavity field is seeded with 1 μW of broadband noise to initiate spontaneous emission processes. Under these conditions, a Q‐switched pulsed laser is established, as shown in **Figure** **3**a. The figure illustrates the temporal variation of the gain coefficient and the intracavity energy, which is the fundamental mechanism underlying Q‐switched pulse generation.

Subsequently, the spectral curves of metafiber‐FP, which serve as trap filters, are integrated into the coupling model, a critical step for implementing pulsed laser central wavelength tuning as reported in reference.^[^ ^41^ ^]^ In pulsed laser systems, the spectral properties in the frequency domain‐including central wavelength and bandwidth‐are fundamentally governed by the characteristics of the gain medium. The energy level structure and gain profile of the gain medium dictate the wavelength range that can be amplified and emitted by the laser. In conventional stable laser configurations, the output wavelength remains nearly constant due to the fixed gain coefficient and saturation behavior of the gain fiber, which maintains the central wavelength within narrow tolerances under steady‐state operation. Here, we introduce a metafiber‐FP that directly influences the laser's longitudinal mode selection and central wavelength within the Er^3 +^ gain bandwidth through modulation of intracavity liquid temperature and RI value. By superimposing these curves onto the gain spectrum (Figure 3b), selective wavelength tuning of the Q‐switched laser is achieved via spectral valley positioning as the center wavelength for Q‐switching pulse simulations. Incorporating the temperature‐ and RI‐dependent reflectance spectra from Figures 2c,d into the model, we numerically characterize the tunable wavelength dynamics of Q‐switched pulses (Figures 3c,d). Systematic tuning of temperature and RI yields wavelength shifts of ∼10 and ∼20 nm, respectively, validating the metafiber‐FP's efficacy as a tunable spectral filter.

### 2.3 Fabrication of Metafiber‐FP

**Figure 4:**
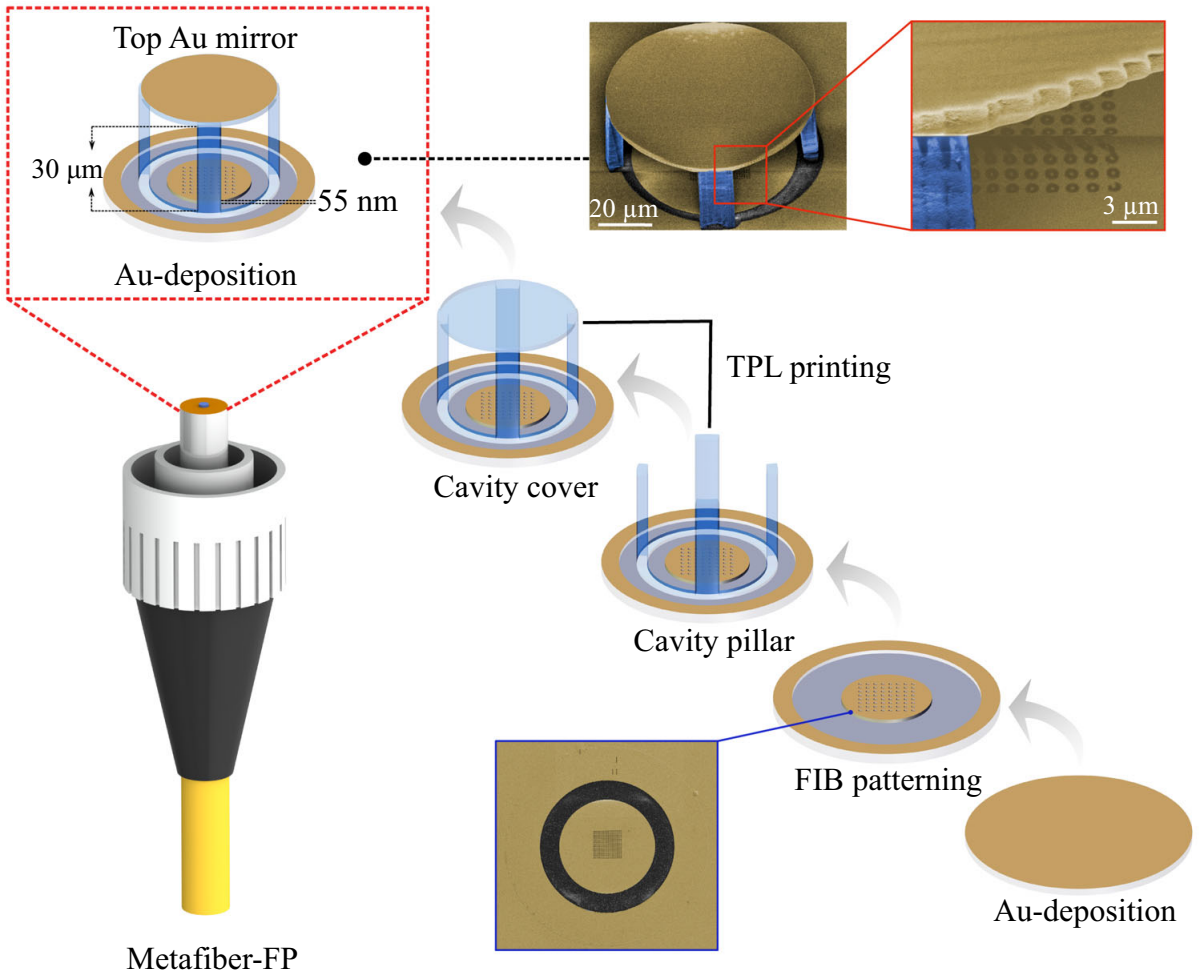
Fabrication flow schematics of the metafiber‐FP. The fabrication flow involves focused ion beam milling for fabricating the nanoeye metasurface; Two‐photon polymerization 3D printing for constructing the FP cavity; PVD for depositing the gold mirror. The pseudocolor SEM images exhibit microscopic configurations of the metafiber and metafiber‐FP, respectively.

To fabricate the metafiber‐FP by integrating 2D metasurfaces and 3D FP cavities at the optical fibers' end facets, we build upon our prior works^[^ ^19^, ^32^ ^]^ to develop a multi‐dimensional fabrication method. **Figure** **4** outlines the processing workflow, which combines planar micro‐nano fabrication using physical vapor deposition (PVD) and focused ion beam (FIB) milling with 3D microfabrication via two‐photon lithography (TPL) at the fiber end facet. The planar fabrication steps for constructing metasurfaces on commercial single‐mode fiber jumper tips including PVD‐deposited 55 nm‐thick gold films and FIB patterning, are detailed in our previous studies.^[^ ^19^ ^]^ As depicted in the bottom panel of Figure 4, a 30 kV, 10 pA Ga^+^ FIB system is used to precisely mill the nanoeye metasurface structure with high precision. The metasurface, featuring an inner diameter of 280 nm, outer diameter of 550 nm, and periodicity of 850 nm is pattered within the single‐mode optical fiber jumper (SMFJ) core region, thereby forming the metafiber component. To circumvent the undesirable defects during the TPL process, a larger microeye structure (60 μm inner diameter, 100 μm outer diameter) is patterned using a 30 kV, 600 pA Ga^+^ FIB system around the pre‐fabricated nanoeye region. Subsequent TPL is performed using a commercial PPGT2 system (Nanoscribe GmbH) above the metafiber structure. Given the SMFJ end‐face integration, we have adapted the fiber holder design^[^ ^42^ ^]^ to accommodate the metafiber's plug‐and‐play functionality, enabling direct mechanical and optical coupling with the TPL setup. The polymer‐based FP cavity was then constructed via layer‐by‐layer TPL printing, with a 30 μm‐height cavity pillar specifically designed to facilitate liquid sample infiltration into the cavity. Detailed TPL parameters and modified fiber holder specifications are provided in our previous work.^[^ ^32^ ^]^ Finally, a top gold film coating was deposited onto the polymer cavity cover, completing the metafiber‐FP hybrid structure. The scanning electron microscopy (SEM) image reveals an intact nanoeye metasurface beneath the FP cavity, demonstrating excellent compatibility between the planar (FIB) and 3D (TPL) fabrication processes on the SMFJ end facet.

### 2.4 Q‐Switched Pulsed Laser Based on Metafiber‐FP

**Figure 5:**
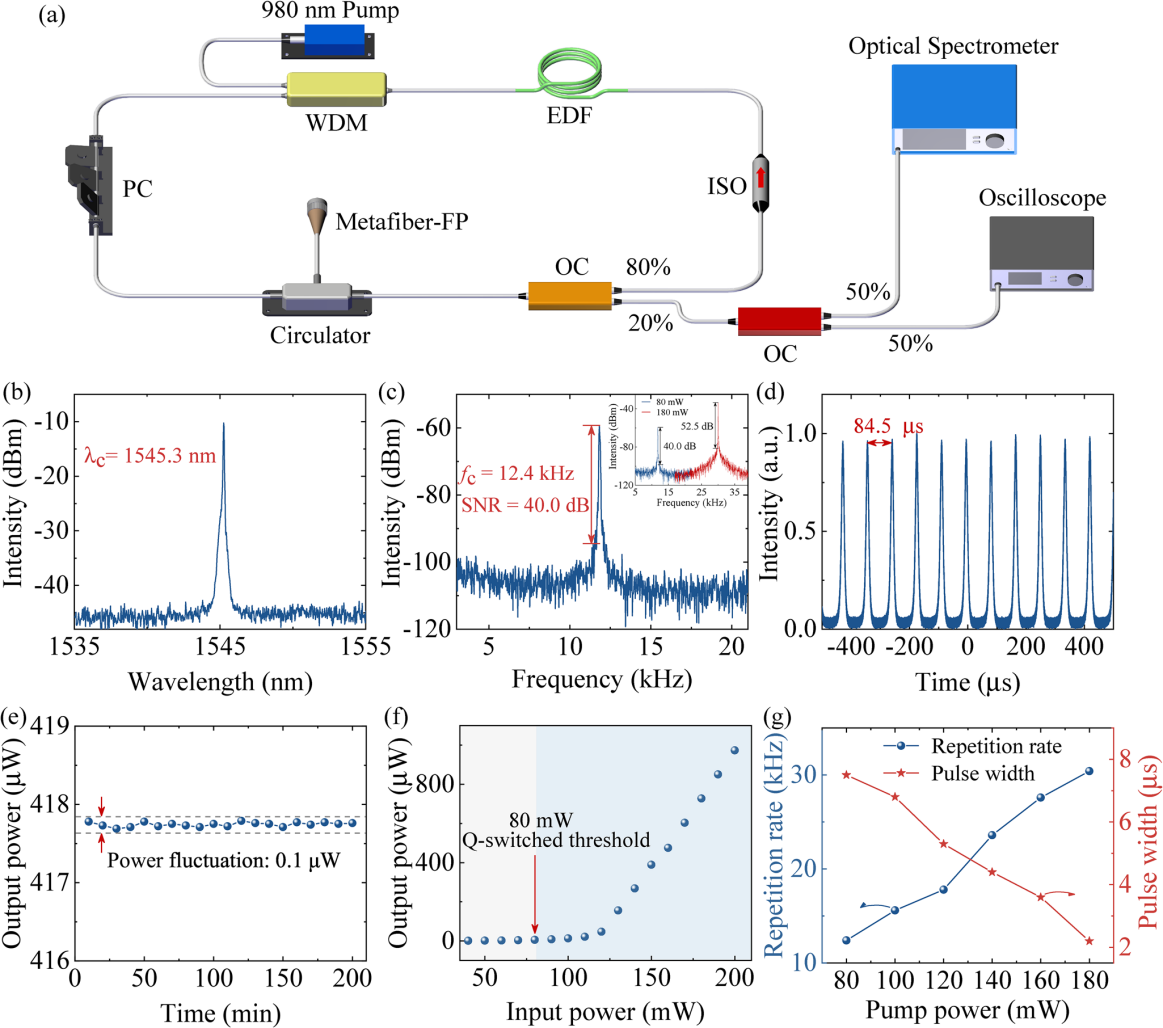
Output characteristics of Q‐switched pulsed lasers under 80 mW pump power. a) Schematic diagram of fiber laser setup. b) Optical spectrum of Q‐switched pulses. c) RF spectrum of Q‐switched pulses. d) Oscilloscope trace of Q‐switched pulses. e) Long‐term stability of a Q‐switched pulses. f) Averaged output power versus pump power. g) Repetition rate and pulse width versus the pump power.

This section presents an experimental demonstration of a Q‐switched laser utilizing a metafiber‐FP filled with a 99.99% purity alcohol solution as an SA. The insertion loss induced by ethanol in the metafiber‐FP structure is only 1%, primarily due to its excellent optical transparency and low absorption properties. This enables the optical signal to maintain a high transmission efficiency when passing through the solution. The laser cavity shown in **Figure** **5**a is meticulously constructed with key components, including a 980 nm pump light source, a wavelength‐division multiplexer (WDM) for beam coupling, an Er‐doped gain fiber for 1550 nm laser generation, a polarization controller (PC), an isolator to maintain unidirectional optical transmission, and an optical coupler (OC) to output 20% of the beam. A circulator is used to connect the metafiber‐FP to the laser cavity^[^ ^43^ ^]^ taking the highly reflective property of the metafiber‐FP into account. Experimental results indicate that Q‐switched pulses commence generation when the pump power surpasses 80 mW. Figures 5b‐d illustrate the performance characteristics of the Q‐switched pulsed laser at a pump power of 80 mW. The optical spectrum exhibits a central wavelength of 1545.3 nm, and the radio frequency (RF) spectrum peaks at 11.8 kHz with a signal‐to‐noise ratio (SNR) of 40 dB at a 100 kHz resolution, which aligns well with the time interval of 84.5 μs measured by the oscilloscope. The pump power is a key parameter for regulating laser performance. An increase in pump power can effectively enhance the stability of Q‐switched pulses, as shown in the inset of Figure 5c. Specifically, with the pump power adjusted from 80 to 180 mW, an SNR improvement of 12.5 dB is achieved. Furthermore, the long‐term power stability of a Q‐switched pulse is demonstrated, as shown in Figure 5e. A slight fluctuation of mere 0.1 μW is recorded when the pump power is maintained for about 200 min. In metafiber‐FP cavity, thermal effects are the dominant stability‐limiting factor. Compared with an air‐filled cavity, the ethanol bath provides an inherent liquid‐cooling mechanism. The liquid's thermal conduction and convection efficiently dissipate heat from the cavity, suppressing temperature build up, sustaining stable output and extending device lifetime. As shown in Figure 5f, the output power curve of the laser shows a Q‐switched threshold the laser shows a Q‐switched threshold of 80 mW; below this value, no stable Q‐switched pulses are generated and the output power remains low. Once the pump power exceeds 80 mW, Q‐switched pulses emerge and the output power rises rapidly. As demonstrated in Figure 5g, by varying the pump power from 80 to 180 mW, the pulse width can be adjusted within the range of 2.2‐7.5 μs, and the repetition frequency can be tuned from 12.4 to 30.4 kHz, showcasing the flexibility and controllability of this Q‐switched laser system. To maintain safe and stable output from the fiber laser, we deliberately avoided higher pump powers during the experiment. High‐energy Q‐switched pulses, together with their tunable pulse width and repetition rate, offer broad application prospects in fields such as laser cleaning, laser micromachining, and biomedical engineering (e.g., ophthalmic and dental laser therapies).^[^ ^44^ ^]^

### 2.5 Temperature Modulation Scheme for Wavelength‐Tunable Pulsed Laser

**Figure 6:**
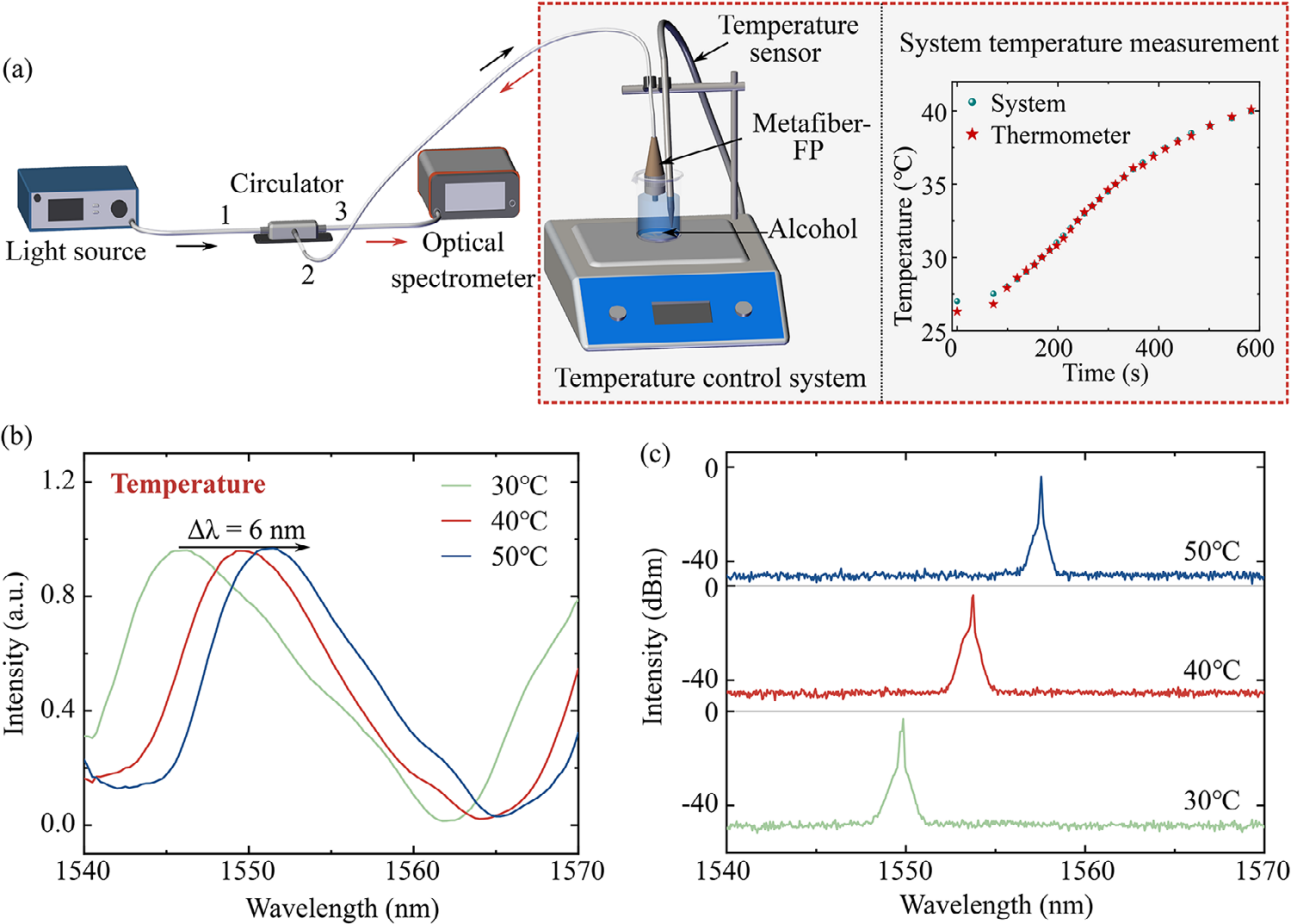
Temperature modulation scheme for wavelength‐tunable Q‐switched pulsed laser. a) Schematic diagram of temperature‐control system. b) Reflectance spectra of the metafiber‐FP under different temperatures. c) Wavelength‐tunable spectra of Q‐switched pulsed laser through the temperature control.

This section focuses on the frequency‐domain spectral modulation enabled by a metafiber‐FP temperature‐control system, as illustrated in **Figure** **6**a. The system is designed to precisely modulate the optical properties of the metafiber‐FP by altering its temperature. The metafiber‐FP is immersed in an alcohol bath, with temperature increments set at 10°C intervals. Temperature control was achieved by a magnetic‐stirrer heating stage with a temperature‐resolution of 0.1°C. To ensure spatial uniformity within the ethanol bath, continuous magnetic agitation was employed: the rotating stir bar efficiently facilitates heat redistribution, mitigating both hot spots and thermal gradients. The accuracy of the integrated sensor was verified by cross‐referencing its readings with an external calibrated thermometer. As shown in the inset of Figure 6a, the two temperature traces overlap almost perfectly. Throughout the heating process, the system exhibits a three‐stage heat “slow‐fast‐slow” profile, which suppresses thermal shock and precludes localized overheating, thereby ensuring stable and reproducible experimental conditions. To prevent ethanol evaporation during the experiment, we covered the beaker with a layer of parafilm to keep the concentration constant over short periods. For long‐term stability, we recommend switching to a sealed vessel such as a GL45‐threaded Erlenmeyer flask fitted with a septum‐lined screw cap. A small hole drilled in the middle of the cap allows the optical fiber to pass through while maintaining an airtight seal. Temperature variations directly impact the optical path length of the FP cavity, which in turn shifts the resonance wavelength of the metafiber‐FP, thereby functioning as a tunable filter for frequency‐domain adjustments. The experimental setup involves injecting the light source into port 1 of a circulator, which then directs the light through port 2 to the metafiber‐FP. The output optical signal is captured by a high‐resolution spectrometer, allowing real‐time spectral monitoring, the processed spectra in the 1.5 μm position as shown in Figure 6b (see details of “Temperature modulation” from Supporting Information). Upon filling the FP cavity with an alcohol solution and increasing the solution temperature, the length of the FP cavity is modulated, altering the free spectral range of the FP structure. This results in enhanced spectral modulation capabilities of the metafiber‐FP, evident from the increased quality factor and expanded modulation range. As temperature increases, the spectral noise floor exhibits a slight decrease, primarily due to thermally induced changes in the metafiber‐FP cavity and the ethanol filling medium, which together alter the resonant conditions and thereby shift the noise baseline. Building upon these findings, the temperature control sub‐system is integrated into the fiber laser system to enable dynamic wavelength modulation of the Q‐switched pulsed laser. By adjusting the temperature of the alcohol bath from 30 to 50°C, continuous tuning of the central wavelength from 1549.0 to 1557.0 nm is achieved, as demonstrated in Figure 6c (see details of “Temperature modulation” from Supporting Information). This showcases the effectiveness of the proposed temperature‐control mechanism in achieving precise spectral modulation for fiber laser applications.

### 2.6 Refractive Index Modulation Scheme for Wavelength‐Tunable Pulsed Laser

**Figure 7:**
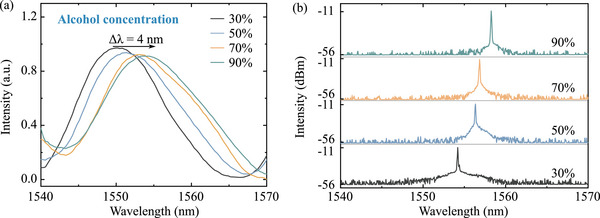
Refractive index modulation scheme for wavelength‐tunable Q‐switched pulsed laser. a) Reflectance spectra of the metafiber‐FP immersed into solution of different RI values. b) Wavelength‐tunable spectra of Q‐switched pulsed laser through the RI control.

The resonance wavelength of the metafiber‐FP is also influenced by the RI value of the alcohol solution filling its cavity. By adjusting the alcohol concentration in an alcohol/water mixture, the RI of the solution can be systematically tuned, leading to observable shifts in the device's reflectance spectra. Ethanol‐water mixture was chosen to modulate the refractive index primarily because this mixture meets the resonant condition for strong resonance within the Er^3 +^ ion gain region.^[^ ^45^ ^]^ This resonance allows wavelength tuning of Q‐switched pulses in the 1.5 μm band: by adjusting the ethanol‐to‐water ratio, we can precisely control the refractive index of the mixture, enabling fine tuning of the laser wavelength. Another reason for selecting ethanol solution is its excellent wettability,^[^ ^46^ ^]^ which ensures uniform filling of the optical cavity and homogeneous coverage on optical component surfaces. This is crucial for maintaining the stability and consistency of the cavity. In contrast, other reagents may exhibit insufficient wettability, leading to non‐uniform filling that can degrade optical performance and compromise experimental reproducibility. As shown in **Figure** **7**a, increasing the alcohol concentration induces a clear red shift in the resonance wavelength of the metafiber‐FP, a direct consequence of the RI‐dependent optical path length within the FP cavity (see details of “Concentration modulation” from Supporting Information). When integrated into the fiber laser system, this RI modulation mechanism enables wavelength tunability of the Q‐switched pulsed laser, as demonstrated in Figure 7b. This optical loss necessitates higher pump power to maintain Q‐switching operation, resulting in spectral pedestals in the output spectra (see Figure 7b), which reflect the energy compensation required for stable pulse generation. Filters, as the key tuning elements in tunable pulsed lasers, have already been extensively studied, as shown in **Table** **1**. Here, we integrate a filter design into the metafiber‐FP structure, enabling the device to simultaneously exhibit saturable absorption and wavelength tunability. The results demonstrate a 10 nm tuning range, which meets the recognized criterion for wavelength‐tunable operation. The wavelength‐tunable pulse‐generation threshold is 80 mW, which is comparable to values reported for other saturable absorbers. However, the energy‐conversion efficiency is only 0.5%, a clear shortcoming of the present system. Future work will focus on minimizing overall cavity losses to improve this efficiency.

**Table 1:** Wavelength‐tunable pulsed laser with different tuning mechanisms.

Tuning mechanism	SA	Integration level	λ (nm)	τ (s)	*f*_*rep*_ (Hz)	Tunable range (nm)	Threshold (mW)	Efficiency	Refs.
Tunable filter	Black phosphorous	2	1060.1	4.21 μ	24.6 k	1054.4‐1068.2	320	2.50%	^[^ ^47^ ^]^
Variable optical attenuator	Carbon nanotubes	5	1570	—	27.5 k	1537.28‐1605.18	12.8	1.56%	^[^ ^48^ ^]^
Mach‐Zehnder filter	Ag nanoparticles	2	1930.0	5.9‐5.1 μ	36.0‐50.1 k	1916.5‐1945.3	176	1.13%	^[^ ^49^ ^]^
Lyot filter	PbS quantum dots	5	1556.6	6.6 n	9.82 M	1550.21‐1560.64	51.4	6.20%	^[^ ^50^ ^]^
Fabry‐Perot filter	Carbon nanotubes	2	—	7.0 μ	15.5 k	1965.4‐2007.35	62.57	6.13%	^[^ ^49^ ^]^
Birefringence filter	Carbon nanotubes	3	1554	243 f	28.3 M	1532‐1562	48	2.22%	^[^ ^51^ ^]^
Pump power	Graphene	2	—	143‐122 n	0.964 M	1897.69‐1930.27	28.8	—	^[^ ^52^ ^]^
Digital micromirrror	Graphene oxide	4	1546.1	7.1‐3.7 μ	43.3‐65.4 k	1528.2‐1559.3	20	—	^[^ ^53^ ^]^
Polarization controller	Bi_2_Te_3_	3	1571	4.5‐6.2 p	10.71 M	1548.2‐1570.1	40	6.33%	^[^ ^54^ ^]^
Grating	MoS_2_	2	1560.5	3.7‐1.92 μ	28.6‐114.8 k	1529.8‐1570.1	41.8	0.36%	^[^ ^55^ ^]^
Metafiber‐FP	Metafiber	1	1545.3	7.5‐2.2 μ	12.4‐30.4 k	1549‐1557	80	0.50%	This work

Note. Integration level refer to the number of devices that can achieve pulse generation and wavelength tunability within a laser resonator.

## 3 Conclusion

To tackle the complexity and alignment challenges in conventional hybrid fiber systems for wavelength‐tunable pulsed laser generation, we propose and demonstrate a metafiber‐FP configuration. This innovative structure integrates a 2D metafiber with a 3D FP interferometer at the end facet of a single‐mode optical fiber, simultaneously serving as both a saturable absorber and a tunable optical filter. By establishing a coupled model that merges the modified nonlinear Schrödinger equation with the rate equation, we achieve a dynamic wavelength‐tuning scheme capable of covering up to 20 nm via the introduced notching filtering effect. Leveraging the optical tunability of the FP cavity, our design enables Q‐switching operation in the telecommunication band and realizes approximately 10 nm dynamic wavelength tuning through temperature/refractive index modulation. This work not only provides a promising approach for pulsed laser frequency‐domain modulation but also expands new frontiers for metasurface technology in fiber photonics, fostering further advancements and innovations in related fields.

## Conflict of Interest

The authors declare no conflict of interest.

## Author Contributions

M.Q., B.F., and L.Z. lead the whole research project. C.Z and L.Z. conceived the main conceptual ideas. L.Z. and W.C. designed the configuration of metafiber. C.Z. designed and carried out the experiments for the laser section. C.Z. and L.Z. analyzed the data and wrote the original manuscript. C.Z., G.W., and L.Z. designed simulation model. Z.Z. and Z.O. contributed to figure production. All authors contributed to the discussion and writing of the manuscript. B.F. and C.Z. contributed equally to the first authors of this work.

## Supporting information

Supporting Information

## Data Availability

The data that support the findings of this study are available from the corresponding author upon reasonable request.
