# Supplementary material for: Wavelength Tunable Pulsed Lasers Enabled by a Versatile Metafiber Functioning as Both Saturable Absorber and Filter
Source: Adv Sci (Weinh). 2025 Oct 17;13(3):e11572. doi: 10.1002/advs.202511572 (PMC12806345; doi:10.1002/advs.202511572)
Supplement: Supplementary file 1 — Supporting Information [file ADVS-13-e11572-s001.docx]

**Wavelength tunable pulsed lasers enabled by a versatile metafiber functioning as both saturable absorber and filter**

**Bo Fu^1,2,*,#^, Chenxi Zhang^2#^, Zhouqi Zhang^2^, Zuxi Ouyang^3,4^, Gang Wang^5^, Xiuhan Jing^2^, Weilin Chen^6^, Lei Zhang^6*^, Min Qiu^3,4,7*^**

^1^International Research Center for Sustainable Photonics, Hangzhou International Innovation Institute, Beihang University, Hangzhou 311115, China.

^2^Key Laboratory of Precision Opto-Mechatronics Technology of Education Ministry, School of Instrumentation and Optoelectronic Engineering, Beihang University, Beijing 100191, China.

^3^Key Laboratory of 3D Micro/Nano Fabrication and Characterization of Zhejiang Province, School of Engineering, Westlake University, 18 Shilongshan Road, Hangzhou 310024, Zhejiang Province, China.

^4^Institute of Advanced Technology, Westlake Institute for Advanced Study, 18 Shilongshan Road, Hangzhou 310024, Zhejiang Province, China.

^5^Photonic Systems Laboratory (PHOSL) École Polytechnique Fédérale de Lausanne (EPFL) Lausanne 1015, Switzerland.

^6^QianYuan National Laboratory, HangZhou, 310000, China.

^7^Westlake Institute for Optoelectronics, Fuyang, Hangzhou 311421, China.

^#^ These authors contributed equally.

**Temperature modulation**

The temperature-control system was designed and constructed as shown in Figure S1a, a detailed description of which has been provided in the main text. Figure S2b depicts the reflection spectra of the metafiber-FP over 1450-1600 nm at various temperatures. The red-shaded region in Figure S1b corresponds to the curve presented in Figure 6b of the main text. In addition to the data in Figure 6c, we also recorded spectra at 35°C and 45°C, as shown in Figure S1c. For direct comparison with the metafiber-FP reflectance spectra, only the curves of 30°C, 40°C and 50°C were retained in the main text.


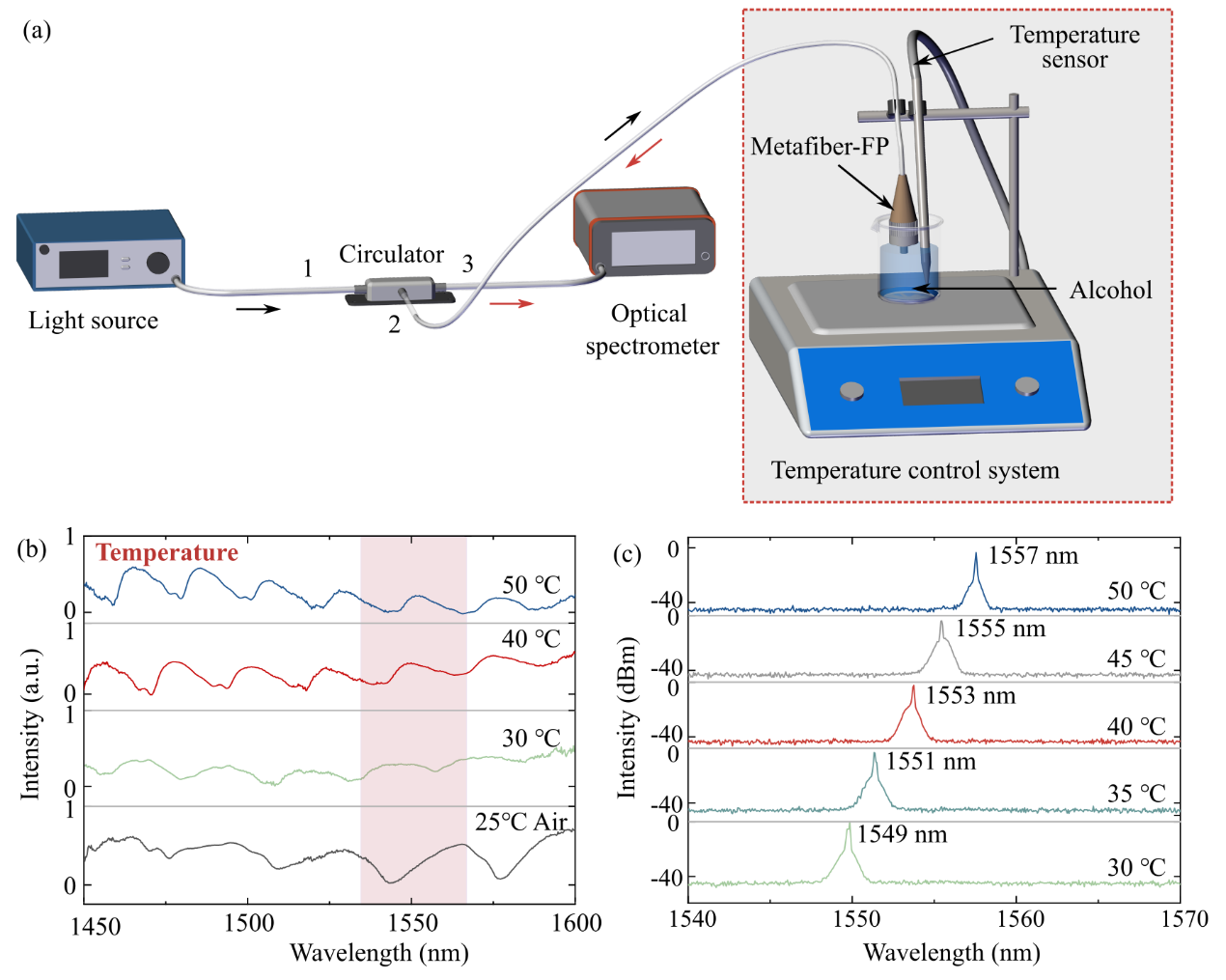


Figure S1: Temperature modulation scheme for wavelength-tunable Q-switched pulsed laser. (a) Schematic diagram of the temperature control system. (b) Reflectance spectra of the metafiber-FP under different temperatures. (c) Wavelength-tunable spectra of Q-switched pulsed laser through temperature control.

**Concentration modulation**

As with the temperature control experiments, reflection spectra of the metafiber-FP under concentration control were acquired over an extended wavelength range of 1450-1600 nm, as illustrated in Figure S2. However, since the center wavelength of Q-switched pulsed laser spectra exhibited a minimal shift with changing concentration, further measurements were not performed.


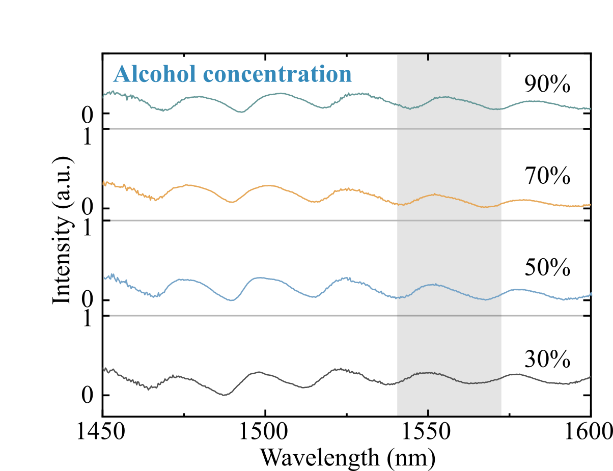


Figure S2: The spectra of Q-switched pulsed laser at different alcohol concentration.
